# HIF-1 regulates pathogenic cytotoxic T cells in lupus skin disease

**DOI:** 10.1172/jci.insight.166076

**Published:** 2023-08-22

**Authors:** Alicia J. Little, Ping-Min Chen, Matthew D. Vesely, Rahanna N. Khan, Jacob Fiedler, James Garritano, Fahrisa I. Maisha, Jennifer M. McNiff, Joe Craft

**Affiliations:** 1 Department of Dermatology and; 2 Department of Immunobiology, Yale University School of Medicine, New Haven, Connecticut, USA.; 3 Institute of Biochemistry and Molecular Biology, National Taiwan University College of Medicine, Taipei City, Taiwan.; 4 Department of Internal Medicine (Rheumatology),; 5 Medical Scientist Training Program, and; 6 Department of Pathology, Yale University School of Medicine, New Haven, Connecticut, USA.

**Keywords:** Autoimmunity, Dermatology, Autoimmune diseases, Lupus, T cells

## Abstract

Cutaneous lupus erythematosus (CLE) is a disfiguring autoimmune skin disease characterized by an inflammatory infiltrate rich in T cells, which are strongly implicated in tissue damage. How these cells adapt to the skin environment and promote tissue inflammation and damage is not known. In lupus nephritis, we previously identified an inflammatory gene program in kidney-infiltrating T cells that is dependent on HIF-1, a transcription factor critical for the cellular and developmental response to hypoxia as well as inflammation-associated signals. In our present studies using a mouse model of lupus skin disease, we find that skin-infiltrating CD4^+^ and CD8^+^ T cells also express high levels of HIF-1. Skin-infiltrating T cells demonstrated a strong cytotoxic signature at the transcript and protein levels, and HIF-1 inhibition abrogated skin and systemic diseases in association with decreased T cell cytotoxic activity. We also demonstrate in human CLE tissue that the T cell–rich inflammatory infiltrate exhibited increased amounts of HIF-1 and a cytotoxic signature. Granzyme B–expressing T cells were concentrated at sites of skin tissue damage in CLE, suggesting relevance of this pathway to human disease.

## Graphical abstract

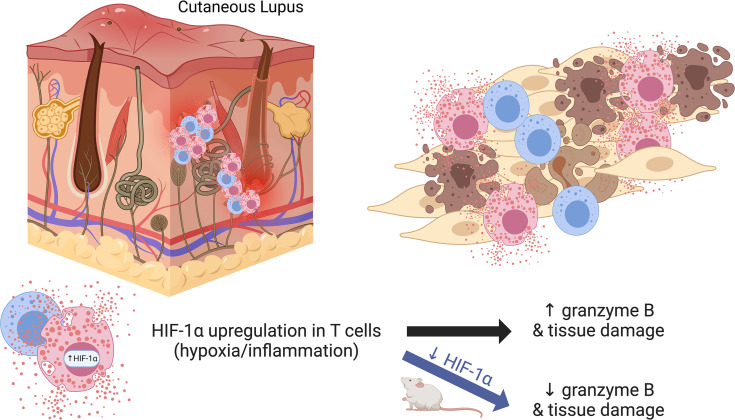

## Introduction

Skin and kidney are among the most frequent targets of organ damage in systemic lupus erythematosus (SLE), and the pathological features include autoantibody deposition and subsequent T cell–mediated tissue injury (1, 2). The changes in environment experienced by T cells as they transition from systemic circulation to local injured tissue likely shape the phenotype and function of T cells upon infiltration into the organ and affect the extent of damage. Indeed, skin-infiltrating myeloid cells from patients with SLE appear to experience such a transition upon migration into the skin, upregulating chemokines and IFN-responsive genes that may result in DC activation and promote local inflammation (3). Similarly, we have demonstrated that oxygen tension deteriorates locally in lupus nephritis, and the subsequent upregulation of the transcription factor HIF-1 dictates the phenotype of kidney-infiltrating T cells and their promotion of tissue damage (4). Blocking HIF-1 by either genetic modification or pharmacological blockade dramatically reduced infiltrating T cell numbers and reversed kidney damage. However, whether HIF-1 plays a role in the development of tissue damage in other organ systems affected in SLE remains unknown.

Cutaneous lupus erythematosus (CLE) may occur as isolated skin disease or may develop in the setting of SLE (5). Similar to the inflammatory infiltrate of lupus nephritis, the CLE inflammatory infiltrate is predominantly composed of T cells (6). It is possible that phenotypic changes in skin-infiltrating T cells in CLE may also occur due to HIF-1 upregulation, but whether this occurs and, if so, how these changes contribute to disease pathogenesis are not known.

HIF-1 plays a critical role in the cellular and developmental responses to hypoxia, as well as in response to inflammation-associated signals including T cell, TLR, and cytokine signaling (7, 8). HIF-1 is a heterodimer composed of HIF-1α (tightly regulated at the transcript and protein levels) bound to HIF-1β (constitutively expressed). In T cells, HIF-1 enhances T cell effector function, including inflammatory cytokine and cytotoxic molecule production (4, 9, 10), and blocks terminal differentiation and exhaustion. HIF-1 also targets Foxp3 for degradation (11), decreasing Treg differentiation and enhancing Th17 development (11, 12).

Under physiological conditions, the oxygen tension in healthy human skin ranges from physiologic (10%, or 76 mmHg) to severely hypoxic (0.1%, or 0.76 mmHg), with the lowest oxygen tension found in the epidermis and portions of some hair follicles and sebaceous glands (13, 14). In CLE, cellular infiltrates are predominantly located in the dermis (6, 15), which contains the dermal vasculature. In healthy skin, this region has physiologic oxygen tension. However, the local oxygen tension in lesional CLE skin is not known, and local tissue damage and inflammation in CLE skin may create a relatively hypoxic microenvironment. In addition, HIF-1 can be upregulated in a hypoxia-independent manner. HIF-1 is accumulated in skin in response to UV light, mediated by mitochondrial ROS (mROS) (16), and HIF-1 is also upregulated in response to several stimuli associated with T cell activation and inflammation, such as T cell receptor, TLR, and cytokine signaling (7), suggesting multiple potential pathways by which HIF-1 might be upregulated in skin-infiltrating T cells.

Based on our prior studies identifying HIF-1 as a key factor for generating pathogenic T cells in lupus nephritis, we sought to determine whether HIF-1 plays a similar role in the development of murine cutaneous lupus-like skin disease (murine CLE). Tissue damage in skin and kidney may share similar mechanisms; however, there are differences in the tissue environments between skin and kidney, and there is apparent stochasticity in development of damage to different organ systems within patients with SLE. To address this knowledge gap and determine whether HIF-1 regulates effector function of skin-infiltrating T cells to cause tissue damage in CLE, we examined the phenotype and function of skin-infiltrating T cells in lesional murine CLE skin as compared with their peripheral T cell counterparts, and we determined the effect of HIF-1 inhibition on skin disease manifestations and skin-infiltrating T cell phenotype. To determine the applicability of our murine findings in the treatment of human disease, we evaluated skin biopsy specimens from patients with discoid lupus erythematosus (DLE), the most common subtype of CLE, for HIF-1 expression and T cell phenotype as compared with healthy human skin.

## Results

**Figure 1:**
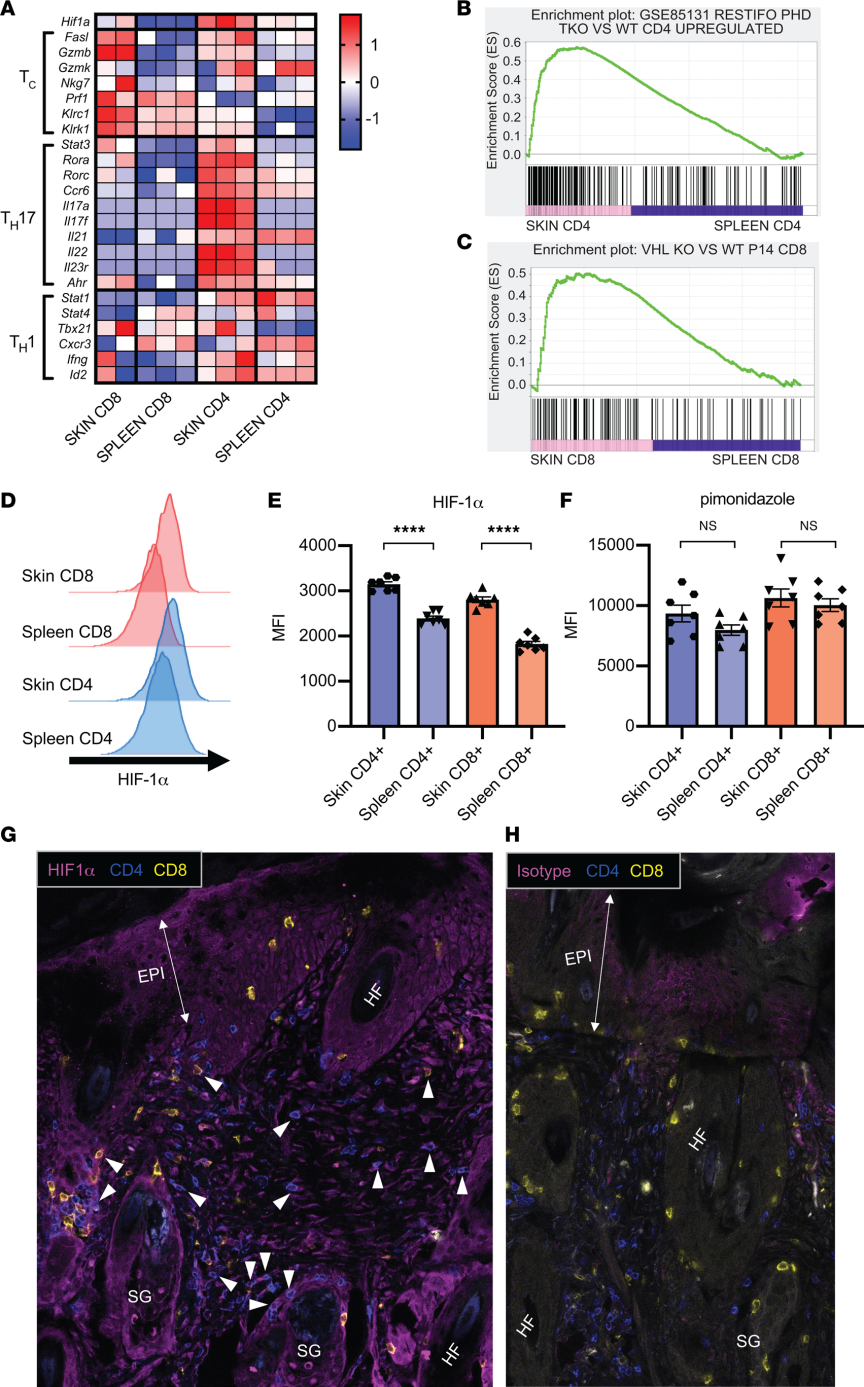
Skin-infiltrating T cells show dominant HIF-1 transcript and protein signatures. (**A**) Heatmap indicating differential expression of cytotoxic T, Th17, and Th1 cell markers between CD4^+^ and CD8^+^ skin-infiltrating and splenic T cells. Data show 2 or 3 biological replicates. (**B**) Gene set enrichment analysis (GSEA) plots comparing gene signatures of skin-infiltrating with splenic CD4^+^ T cells based upon the hypoxia signature generated by comparing triple prolyl-hydroxylase–knockout (PHD-KO) CD4^+^ with WT CD4^+^ T cells (17). (**C**) GSEA plots comparing gene signatures of skin-infiltrating with splenic CD8^+^ T cells based upon the hypoxia signature generated by comparing VHL tumor suppressor KO CD8^+^ T cells with lymphocytic choriomeningitis virus–specific P14 T cell receptor transgenic CD8^+^ T cells taken from virally infected mice (9). (**D** and **E**) Representative data and summary of HIF1α staining of splenic versus skin-infiltrating CD4^+^ and CD8^+^ T cells isolated from the skin of MRL/lpr mice 20 to 22 weeks old. (**F**) Pimonidazole staining of splenic vs. skin-infiltrating CD4^+^and CD8^+^ T cells isolated from the skin of MRL/lpr mice 20 to 22 weeks old. Representative of 2 experiments, *n* = 6–7 animals per group. (**G** and **H**) IF images of MRL/lpr diseased skin stained for HIF1α (**G**) or isotype control (**H**) (magenta), CD4 (blue), and CD8 (yellow). White arrowheads indicate HIF-1–expressing CD4^+^ or CD8^+^ T cells. Representative of 7 total sections from 2 mice (see Supplemental Figure 2). Data shown are mean ± SEM. Statistical analysis by 2-tailed paired Student’s *t* test (**E** and **F**). ****P < 0.0001. EPI, epidermis; SG, sebaceous gland; HF, hair follicle.

To examine phenotypes specific to skin-infiltrating T cells, we sorted CD4^+^ and CD8^+^ T cells from lesional dorsal skin of 20-week-old MRL/MpJ-*Fas^lpr^*/J (MRL/lpr) SLE-prone mice, separating the tissue-resident cells from circulating cells (Supplemental Figure 1; supplemental material available online with this article; https://doi.org/10.1172/jci.insight.166076DS1). We simultaneously sorted CD4^+^ and CD8^+^ T cells from the spleens of these same mice and performed bulk RNA-Seq, comparing skin-infiltrating T cells to their splenic counterparts (Figure 1A) to enable identification of transcriptional programs specifically upregulated in skin-infiltrating T cells from murine CLE skin.

### Skin-infiltrating T cells show a dominant HIF-1 signature at the transcript and protein levels.

As compared with splenocytes, skin-infiltrating CD4^+^ and CD8^+^ T cells demonstrated significantly upregulated *Hif1a* transcript (Figure 1A, top row). In addition to the upregulation of *Hif1a* transcript, gene set enrichment analysis (GSEA) of skin-infiltrating T cells as compared with splenic T cells revealed a dominant HIF-1 signature, as determined by comparison with previously identified sets of genes that are upregulated in the setting of HIF-1 overexpression in CD4^+^ (17) or CD8^+^ T cells (9) (Figure 1, B and C). Thus, in skin-infiltrating CD4^+^ or CD8^+^ T cells from murine CLE skin, we found upregulation of both *HIF1a* transcripts and genes known to be activated by HIF-1 overexpression, as compared with splenic CD4^+^ or CD8^+^ T cells from the same animals.

The upregulation of HIF-1, as identified by RNA-Seq, was confirmed by flow cytometry. HIF-1α protein was more highly expressed in both CD4^+^ and CD8^+^ skin-infiltrating T cells than in their counterparts taken simultaneously from spleens of MRL/lpr mice (Figure 1, D and E). The presence of nuclear HIF-1α was verified by immunofluorescence (IF) staining in skin-infiltrating CD4^+^ and CD8^+^ T cells in lesional interscapular MRL/lpr skin (Figure 1, G and H, and Supplemental Figure 2, A–D). To determine if upregulation of HIF-1α in skin-infiltrating T cells resulted from local hypoxia, pimonidazole (Hypoxyprobe), which binds to thiol groups contained in proteins and peptides at oxygen tensions below 10 mmHg (18) and is used to identify moderate to severely hypoxic conditions (14), was injected into mice before sacrifice. Unlike what was observed in renal-infiltrating T cells (4), the local microenvironment of skin-infiltrating T cells was not demonstrably hypoxic when evaluated by staining for pimonidazole; the difference in pimonidazole staining between skin-infiltrating and splenic T cells was not significantly different (Figure 1F). Despite identification of hypoxic areas in the dermis of diseased skin as identified by pimonidazole staining (Supplemental Figure 3, A–C), a substantial amount of T cell infiltration was also seen in regions without increased pimonidazole staining (Supplemental Figure 3D), consistent with our flow cytometry findings (Figure 1F). These data demonstrate upregulation of HIF-1α in T cells in murine CLE skin, which may be the result of nonhypoxic mechanisms rather than the direct response to hypoxia. However, it is also possible that diseased skin experiences decreased oxygen tension, resulting in modest hypoxia (oxygen partial pressure of ~19 mmHg) (14) that is not detectable using pimonidazole, which requires extremely low oxygen tension (<10 mmHg O_2_) to form the stable thiol adducts that are detected (18).

### Skin-infiltrating T cells demonstrate a cytotoxic and Th17 phenotype.

**Figure 2:**
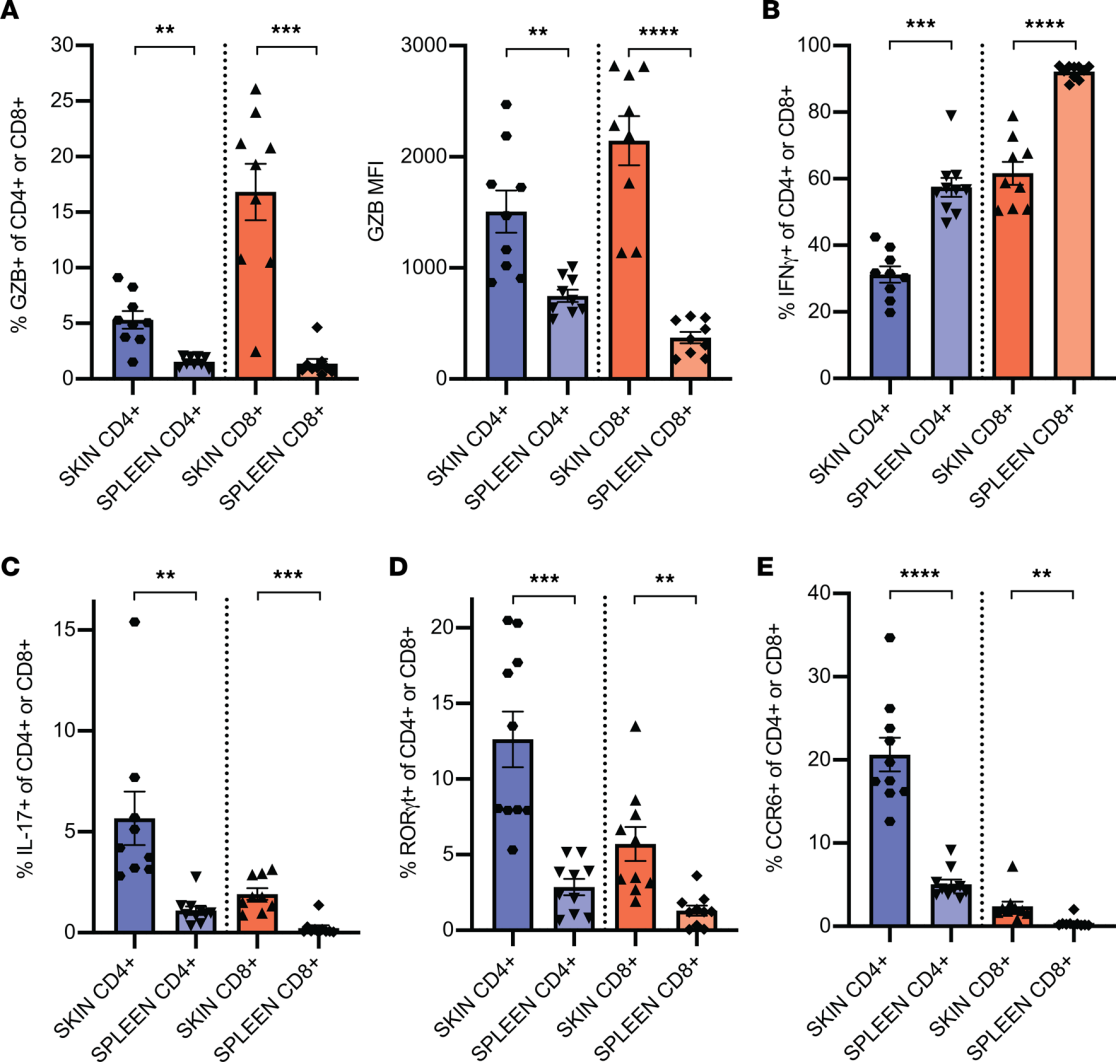
Skin-infiltrating T cells demonstrate activated cytotoxic and Th17 phenotypes. (**A**) Percentage of positive (left) and MFI (right) of granzyme B^+^ (GZB^+^) of activated (CD44^hi^) skin-infiltrating vs. splenic CD4^+^ and CD8^+^ T cells isolated from 20- to 22-week-old MRL/lpr mice after stimulation with PMA/ionomycin for 4 hours after isolation. (**B**) Percentage of IFN-γ^+^ cells of CD44^hi^ skin-infiltrating or splenic CD4^+^ and CD8^+^ T cells after stimulation, as in **A**. (**C**) Percentage of IL17^+^ cells of CD44^hi^ skin-infiltrating or splenic CD4^+^ and CD8^+^ T cells after stimulation as in **A**. (**D**) Percentage of RORγt^+^ cells of CD44^hi^ skin-infiltrating vs. splenic CD4^+^ and CD8^+^ T cells isolated as in **A**. (**E**) Percentage of CCR6^+^ cells of CD44^hi^ skin-infiltrating vs. splenic CD4^+^ and CD8^+^ T cells isolated as in **A**. *n* = 8–10 mice in 2 experiments. Data are mean ± SEM. Statistical analysis by 2-tailed paired Student’s *t* test. **P < 0.01, ***P < 0.001, ****P < 0.0001.

RNA-Seq experiments identified that both skin-infiltrating CD4^+^ and CD8^+^ T cells from murine CLE possessed strong cytotoxic capacity with high *Fasl* and *Gzmb* expression (Figure 1A, labeled T_c_). In addition, skin CD4^+^ T cells demonstrated a strong Th17 transcriptional signature but not a Th1 phenotype (Figure 1A, labeled T_h_17 and T_h_1). These transcriptional results were further assessed by flow cytometric analyses, which confirmed T cell cytotoxic functionality and a dominant Th17 phenotype. As compared with splenic T cells, skin CD8^+^, and to a lesser extent CD4^+^ T cells, expressed significantly more intracellular granzyme B than did their splenic counterparts (Figure 2A). In contrast, the percentages of IFN-γ^+^ skin CD4^+^ and CD8^+^ T cells after stimulation were significantly lower than that of splenocytes (Figure 2B). We also verified the dominant Th17 phenotype in murine CLE skin. A significantly greater fraction of skin-infiltrating CD4^+^ T cells produced IL-17A after ex vivo stimulation, as compared with splenic CD4^+^ T cells (Figure 2C). Furthermore, more skin-infiltrating CD4^+^ T cells expressed the canonical Th17 transcription factor RORγt (Figure 2D) and Th17-associated chemokine receptor CCR6 (Figure 2E), as compared with splenic T cells; in contrast, only a very small fraction of skin-infiltrating CD8^+^ T cells expressed IL-17A or Th17 phenotypic markers (Figure 2, C–E). These results further validate the predominant Th17 phenotype in skin-infiltrating CD4^+^ T cells in murine CLE.

### HIF-1α inhibition abrogates cutaneous disease in association with reduced cytotoxic T cell activity in diseased MRL/lpr skin.

To determine the effect of HIF-1 blockade on preventing skin disease, a 4-week treatment using a selective pharmacologic HIF-1α inhibitor was given to 16-week-old MRL/lpr mice. We have previously shown that this treatment in younger (10- to 12-week-old) MRL/lpr mice decreases systemic disease, as measured by spleen size and anti-dsDNA production, and lupus nephritis, as measured by proteinuria, histopathology, and renally infiltrating T cells (4). However, murine CLE skin lesions may not develop in MRL/lpr mice until after 14–16 weeks of age, and thus our prior experiments did not allow for examination of the effect of HIF-1 blockade on murine CLE.

**Figure 3:**
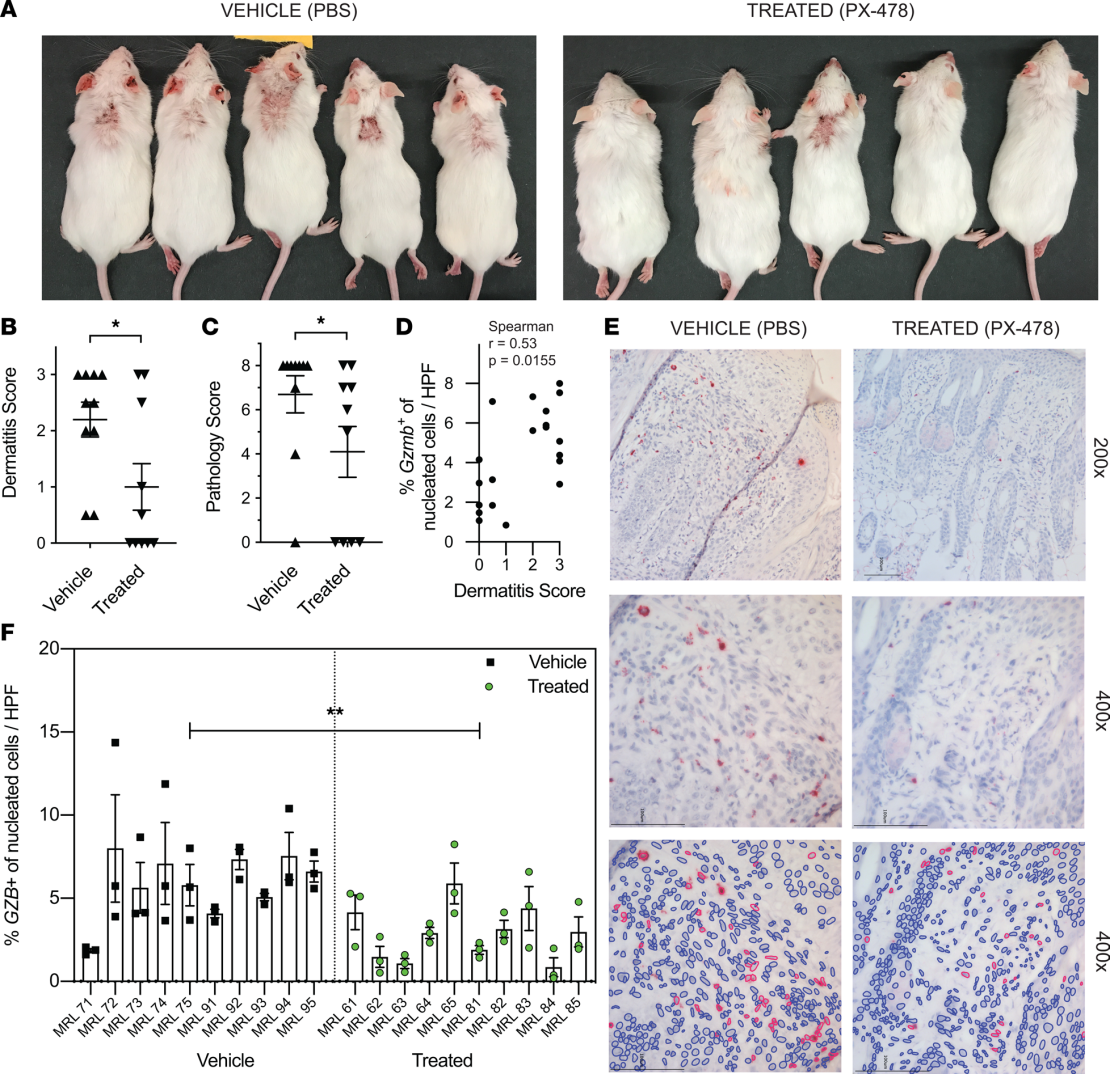
HIF1α inhibition abrogates cutaneous disease and reduces cytotoxic activity in diseased MRL/lpr skin. (**A**) Representative images of clinical disease in 20-week-old MRL/lpr mice after 4 weeks of treatment with either PX-478 or PBS. (**B**) Dermatitis scores at 20 weeks for mice treated as in **A** (*n* = 10 for each group). (**C**) Histopathology damage score (see Methods) of diseased interscapular skin at age 20 weeks for mice treated as in **A** (*n* = 10 for each group). (**D**) Association between clinical disease (dermatitis score) and percentage *Gzmb*^+^ cells per high-power field (HPF) by RNA ISH in 20-week-old MRL/lpr mice treated as in **A**. (**E**) Representative images of RNA ISH for *Gzmb* in 20-week-old MRL/lpr mice after 4 weeks of treatment with either PBS (vehicle) or selective HIF1a inhibitor PX-478 (treated). (**F**) Quantification of percentage *Gzmb*^+^ cells per HPF by RNA ISH in 20-week-old MRL/lpr mice treated as in **A** (3 HPFs per mouse; *n* = 10 mice for each group). Data shown are mean ± SEM. Statistical analysis by 2-tailed Mann-Whitney test (**B** and **C**), Spearman’s correlation (**D**), or nested, 2-tailed unpaired Student’s *t* test (**F**). *P < 0.05, **P < 0.01, ***P < 0.001.

As compared with mice receiving PBS vehicle control, those that received PX-478, a selective HIF-1α inhibitor, had significantly reduced clinical skin disease (according to dermatitis score) at 20 weeks (Figure 3, A and B). In addition, histopathologic disease score (see Methods) of the interscapular skin was reduced in HIF-1α inhibitor–treated mice as compared with vehicle-treated mice (Figure 3C). To identify a potential mechanism by which HIF-1 promotes pathogenic T cells in murine CLE, we evaluated the expression of granzyme B in skin-infiltrating cells in PX-478 or vehicle-treated MRL/lpr mice. Granzyme B expression was positively correlated with severity of clinical skin disease (according to dermatitis score) among both PX-478– and vehicle-treated mice (Figure 3D). PX-478 treatment not only decreased clinical skin disease severity and histopathologic disease score but also decreased the proportion of cells expressing granzyme B in affected skin (Figure 3, E and F). These data together suggested that HIF-1α inhibition may abrogate murine CLE by decreasing T cell cytotoxic activity.

### HIF-1α inhibition reduces cytotoxic activity in CD8^+^ T cells isolated from MRL/lpr CLE skin.

**Figure 4:**
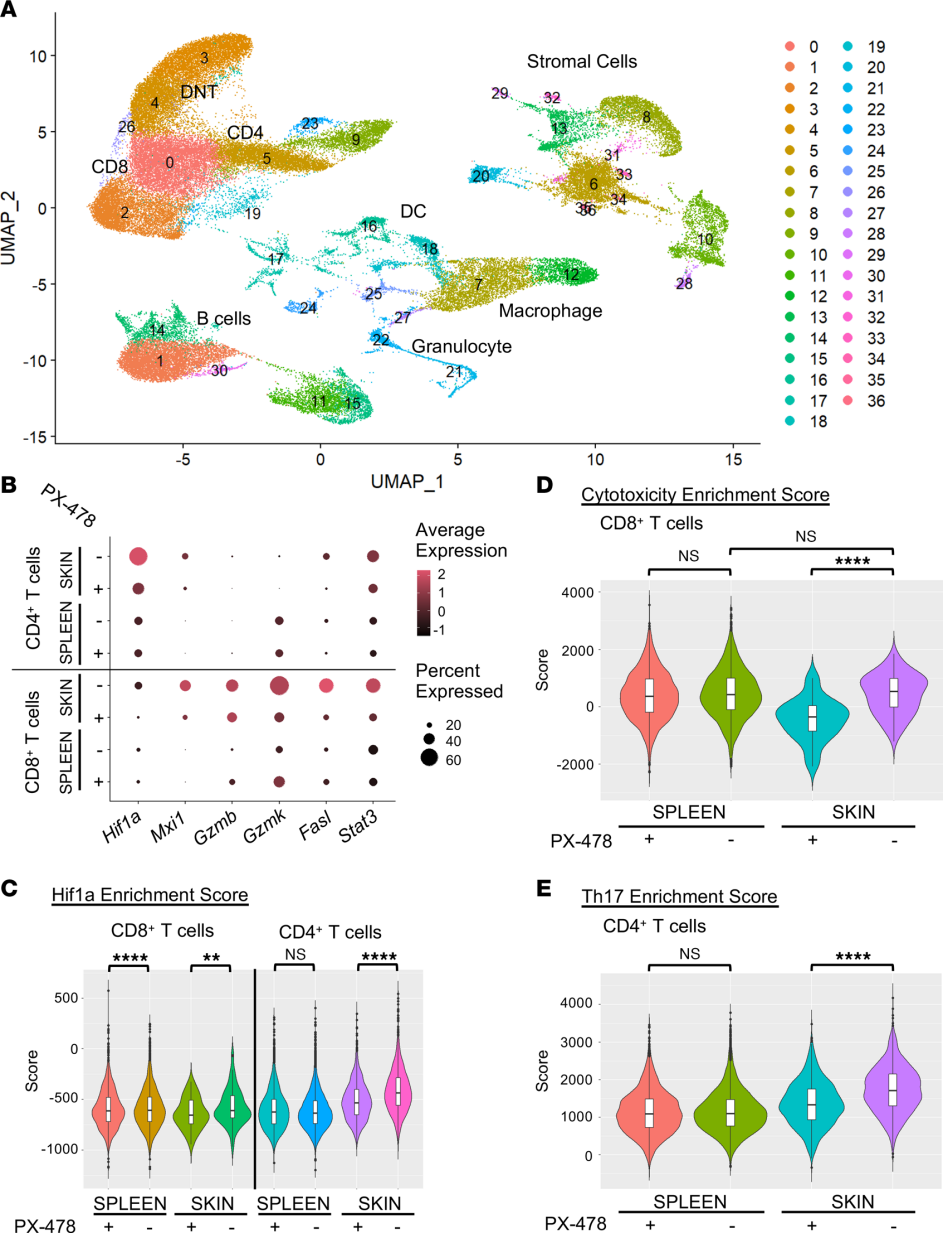
HIF1α inhibition reduces *Hif1a* and cytotoxic activity in CD8^+^ T cells isolated from diseased MRL/lpr skin. (**A**) Combined uniform manifold approximation and projection (UMAP) plot of single-cell RNA-Seq data derived from cells isolated from the skin or spleen of 20-week-old MRL/lpr mice after 5 days of treatment with either PBS (vehicle) or selective HIF1a inhibitor PX-478 (treated). Cluster identities were determined using the cluster identity predictor (CIPR) package in *R* and verified by manual review of cell type–specific transcripts by cluster (see Methods). (**B**) Dot plot demonstrating normalized expression level (average expression) and percentage of cells expressing selected genes in CD4^+^ or CD8^+^ T cell clusters, separated by organ of origin (skin vs. spleen) and treatment group (PBS vs. PX-478). (**C**) HIF1A enrichment score for CD8^+^ (left) and CD4^+^ (right) T cells, separated by organ of origin and treatment group. (**D**) Cytotoxicity enrichment score for CD8^+^ T cells, separated by organ of origin and treatment group, as in **C**. (**E**) Th17 enrichment score for CD4^+^ T cells, separated by origin of cells and treatment group, as in **C**. Pooled data from *n* = 4 mice per treatment group. Statistical analysis by Kruskal-Wallis test. **P < 0.01, ****P < 0.0001.DNT, double-negative T cells; SPL, spleen.

To confirm the effect of pharmacologic HIF-1α blockade on cytotoxic activity in skin-infiltrating T cells, we performed single-cell RNA-Seq of cells isolated from murine CLE skin and spleens taken simultaneously from MRL/lpr mice treated with a 5-day course of high-dose PX-478 or PBS vehicle control. The single-cell suspension made from skin was enriched for hematopoietic (CD45^+^) cells to allow for comparison of skin-infiltrating CD4^+^ and CD8^+^ T cells with their splenic counterparts. Clustering of spleen and skin cells revealed populations of CD4^+^ and CD8^+^ T cells from both spleen and skin, with an expanded, double-negative T cell population identified only in splenocytes; stromal cells, including endothelial cells, fibroblasts, and keratinocytes, were identified only in skin samples (Figure 4A and Supplemental Figure 4). As anticipated, selective pharmacologic HIF-1α inhibition decreased transcripts of *Hif1a* and *Mxi1*, a gene directly regulated by HIF-1α (19), in both CD4^+^ and CD8^+^ skin-infiltrating T cells but not splenic T cells (Figure 4B). This was verified using GSEA, comparing gene expression in CD4^+^ and CD8^+^ T cell clusters against a previously identified set of genes controlled by HIF-1α (20). GSEA demonstrated that HIF-1 inhibitor treatment reduced Hif1α enrichment score in both CD4^+^ and CD8^+^ skin-infiltrating T cells (Figure 4C). HIF-1 inhibition also reduced cytotoxic potential in CD8^+^ skin-infiltrating, but not splenic, T cells, as demonstrated by decreased *Gzmb*, *Gzmk,* and *Fasl* transcripts (Figure 4B), and reduced cytotoxicity enrichment score in skin-infiltrating CD8^+^ T cells (Figure 4D), indicating an organ-specific effect of HIF-1 inhibition on T cell cytotoxicity in the skin, supporting a causal role for HIF-1 in promoting pathogenic cytotoxic T cell activity in murine CLE. HIF-1 inhibition reduced expression of exhaustion markers *Pdcd1* and *Tigit* in skin-infiltrating CD8^+^ T cells (Supplemental Figure 4F); however, the functional consequence of these transcriptional changes is not known. HIF-1α inhibition also reduced Th17 activity in CD4^+^ T cells, as demonstrated by a decrease in Th17 enrichment score in skin-infiltrating CD4^+^ T cells, which was not seen in splenic T cells (Figure 4E), thus identifying a potential role for HIF-1 in promoting pathogenic Th17 T cell activity in murine CLE.

**Figure 5:**
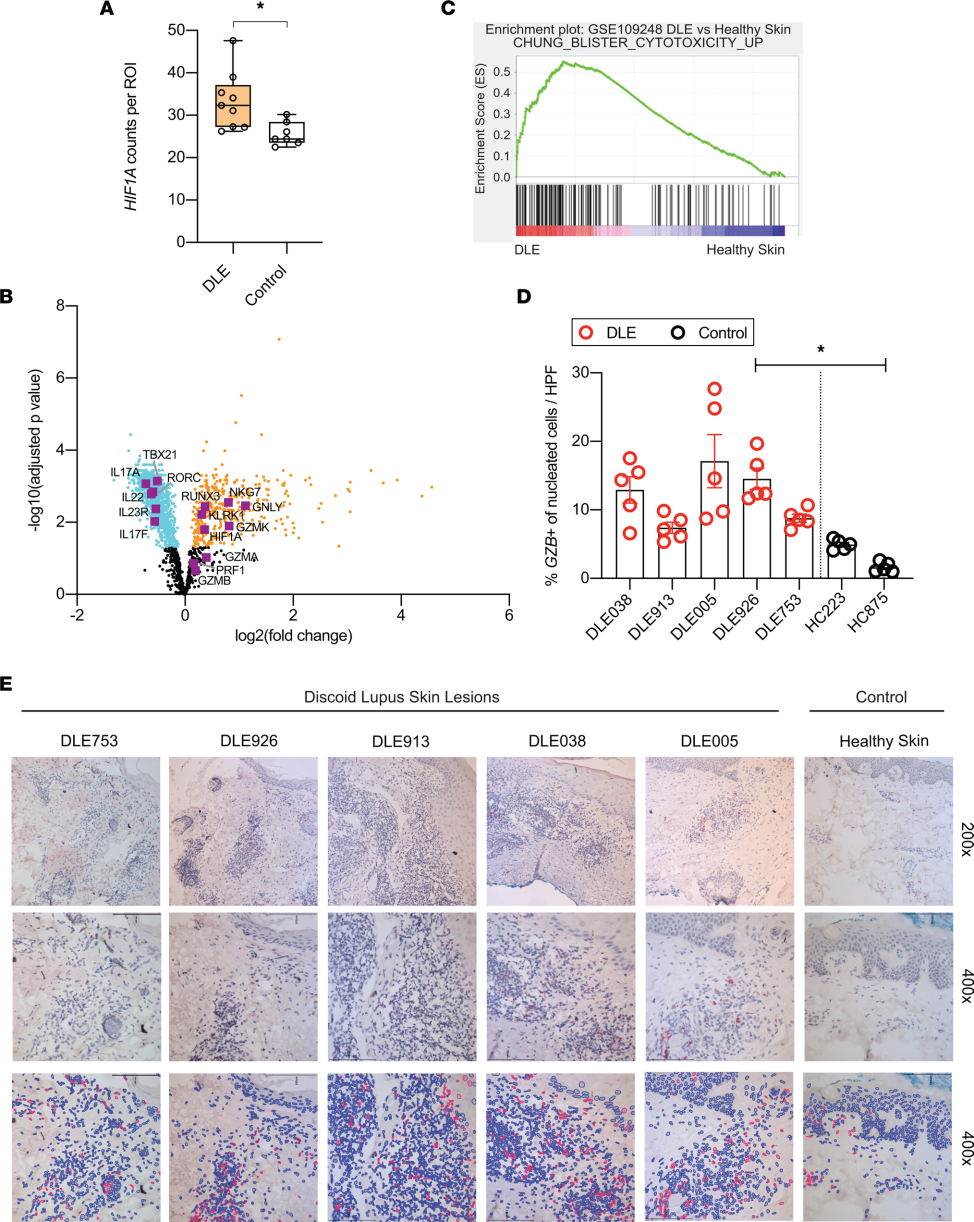
Human DLE demonstrates expression of HIF1α and cytotoxic molecules, including granzyme B, in the DLE lymphocytic infiltrate. (**A**) Normalized *HIF1A* expression per ROI in DLE or normal control skin (*n* = 3 patients; *n* = 3 ROIs per patient), as characterized by NanoString GeoMx Digital Spatial Profiling. ROIs were selected to include T cell–rich infiltrate. (**B**) Volcano plot of NanoString DSP transcripts significantly (*P* < 0.05) upregulated (orange), downregulated (cyan), or not different (black; *P* > 0.05) in DLE vs. healthy control skin. Genes of interest are highlighted in purple. (**C**) GSEA comparing gene signatures from 6 DLE samples with 14 healthy skin samples (GSE109248) (25) based on the cytotoxicity signature generated from comparison of blister fluid with PBMCs from patients with Stevens–Johnson syndrome/toxic epidermal necrolysis (26). (**D** and **E**) Representative data and summary of granzyme B (red) RNA ISH in FFPE DLE and healthy skin detected by RNAScope-RED Assay, ×200 and ×400 original magnification (top, middle panels). Quantified with QuPath (lower panel) (*n* = 5 DLE, *n* = 2 healthy control samples). Statistical analysis by nested, unpaired, 2-tailed *t* test. *P < 0.05.

We next determined whether the HIF-1*,* Th17, and cytotoxic transcriptional signatures that were upregulated in murine CLE were similarly enhanced in skin biopsy specimens from patients with DLE, the most common subtype of CLE. We first analyzed transcriptional profiles of regions of interest selected to include T cell–rich infiltrates using NanoString GeoMx Digital Spatial Profiling (DSP), which identified upregulated *HIF1A* in DLE skin as compared with healthy control skin (Figure 5A). Our findings were in line with a recent microarray study of CLE that identified *HIF1A* network upregulation (21). Our NanoString data also revealed upregulation of cytotoxic signature molecules, including *NKG7*, *RUNX3*, *KLRK1*, and *GZMK* (Figure 5B), confirming that the cytotoxic signature identified in murine CLE-like skin is present in human DLE, consistent with prior literature identifying upregulation of cytotoxic-associated genes in the latter condition (22). Unlike murine CLE, we did not identify a Th17 signature in human DLE skin as compared with healthy skin. Instead, Th17-associated genes *IL17A*, *IL22*, *IL23R*, *IL17F*, and *RORC* were more highly expressed in T cell–rich regions of healthy skin as compared with DLE skin (Figure 5B), consistent with multiple prior studies that identified minimal Th17 activity in human DLE skin (22–24). We further confirmed the robust cytotoxic signature found in DLE skin samples as compared with healthy control skin by performing GSEA, comparing gene signatures from a publicly available microarray dataset of 6 DLE and 14 healthy skin samples (25) against a human skin cytotoxicity signature generated from comparison of blister fluid with PBMCs in patients with Stevens–Johnson syndrome/toxic epidermal necrolysis (26) (Figure 5C).

**Figure 6:**
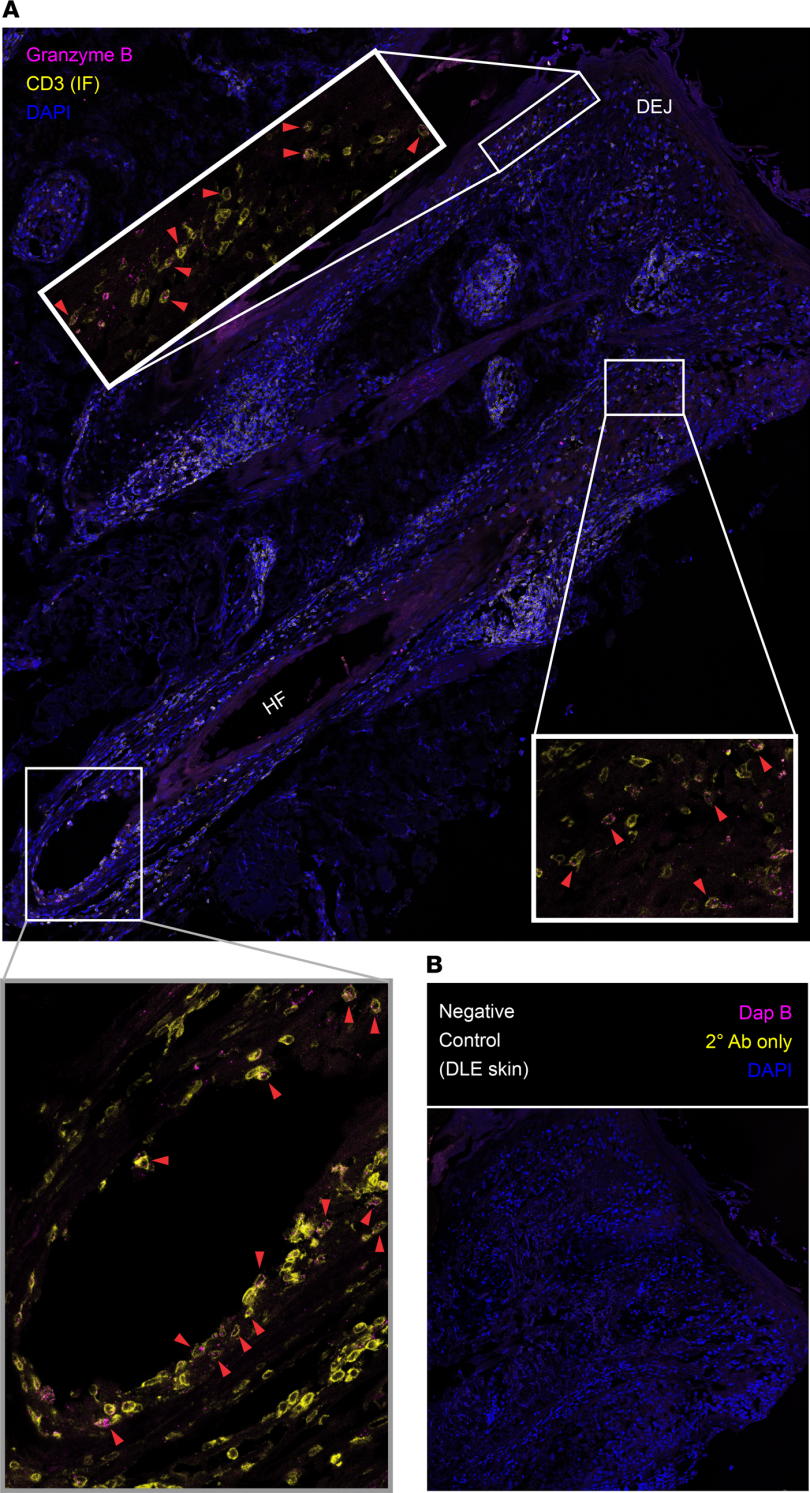
T cells in human DLE express granzyme B at sites of skin tissue damage. (**A**) RNA FISH with simultaneous IF protein detection (FISH-IF) shows granzyme B (pink) transcript in T cells expressing surface CD3 (yellow) in FFPE DLE skin with DAPI-stained nuclei (blue) detected by RNAScope Multiplex Fluorescent V2 Assay at ×400 original magnification (top panel), with enlarged insets of infiltrates at the DEJ and perifollicular areas identifying *GZMB*^+^CD3^+^ T cells (red arrowhead); insets shown without DAPI for clarity. (**B**) RNA FISH-IF negative control in FFPE DLE skin stained for bacterial transcript *Dap B* (pink), secondary Ab only (yellow), and DAPI-stained nuclei (blue). Representative of 3 cases of DLE. HF, hair follicle.

Finally, we evaluated skin biopsy specimens from patients with DLE for granzyme B expression by RNA ISH, as we did previously for MRL/lpr lesional skin (Figure 3). Human DLE skin lesions demonstrated elevated expression of *GZMB* RNA as compared with healthy human skin (Figure 5, D and E), with *GZMB*^+^ cells concentrated in the T cell–rich inflammatory infiltrate (Supplemental Figure 5A). To specifically identify *GZMB*^+^ T cells in DLE lesional skin, we performed IF staining for CD3 with concurrent RNA FISH to *GZMB* transcripts, identifying numerous *GZMB*^+^ T cells at the dermoepidermal junction (DEJ) and perifollicular regions (Figure 6A) as compared with control-stained tissue (Figure 6B). In the majority of cases analyzed, spatial quantification revealed enrichment of *GZMB*^+^ T cells at the DEJ and adjacent to the hair follicle (Supplemental Figure 5, B–E), which represent sites of skin tissue damage in DLE (27).

## Discussion

We identified HIF-1 as a key factor in generating pathogenic T cells in lupus skin disease in lupus-prone MRL/lpr mice, confirming it regulates effector function of skin tissue–infiltrating T cells as we found in kidney-infiltrating T cells in lupus nephritis (4). CLE skin–infiltrating T cells demonstrated increased effector function by transcript and protein expression as compared with their splenic counterparts. Cytotoxic effector function was decreased after systemic pharmacologic HIF-1 inhibition, which also ameliorated the development of skin lesions and skin histopathology score, suggesting that pathogenic cytotoxic effector function may be a consequence of the upregulated HIF-1 signature in skin-infiltrating T cells. HIF-1 is known to drive a transcriptional program that is linked to pathogenic features with enhanced T cell effector function (9, 11), indicating it drives pathogenic T cell activity in CLE. HIF-1 expression and cytotoxic effector capacity were also increased in human DLE skin as compared with healthy skin, suggesting the applicability of its therapeutic blockade to treat human DLE.

Cytotoxic CD8^+^ T cells expressing granzyme B are substantially increased in CLE skin biopsy specimens (28–30) and drive the pathogenic process by promoting cellular apoptosis in the epidermis and papillary dermis (29), although the relative contribution of cytotoxic T cells to the development of tissue damage in skin is not known. Nor is it known whether cytotoxic CD4^+^ T cells, whose granzyme B secretion may be less tightly regulated than that of their CD8^+^ counterparts (31), play a role in CLE pathogenesis. A recent study using single-cell RNA-Seq of skin biopsy specimens from patients with SLE and from healthy control participants did not find elevated activation, cytotoxicity, or exhaustion profiles in T cells from lesional skin (24). However, these results may not accurately characterize the DLE lesional T cell infiltrate, due to the limited number of T and NK cells isolated from lesional skin (*N* = 687 cells); furthermore, only 3 of the 7 patients with SLE who were studied had DLE. Another recent study of single-cell RNA-Seq data from skin biopsy specimens of patients with DLE, SLE or healthy control participants captured more than 40,000 dermal T cells, of which cytotoxic T lymphocytes were a major subset identified. The majority of cytotoxic T cells originated from DLE skin biopsy specimens as compared with SLE or healthy control skin biopsy specimens (32). Using multiple techniques, we found that T cell–rich areas of human DLE skin demonstrated upregulated HIF-1 and a cytotoxic signature, and our murine studies suggested that HIF-1 inhibition abrogated murine CLE by directly decreasing T cell cytotoxic activity, thus ameliorating tissue damage. These findings were in line with prior studies demonstrating that HIF-1 prevents exhaustion in CD8^+^ T cells and promotes granzyme B production (9, 10), and were consistent with prior studies in MRL/lpr splenocytes demonstrating reduced granzyme B production after treatment with HIF-1a shRNA (4). It is also possible that prevention of tissue damage in the setting of HIF-1 inhibition is due to reduced T cell survival in the skin microenvironment, as we identified for the kidney microenvironment on its promotion of lupus nephritis (4).

In addition to CD8^+^ T cells, certain subsets of CD4^+^ T cells, including Th1 and Th17 cells, are increased in some studies of human DLE skin (6). Our studies did not identify a strong Th1 signature in mouse or human DLE skin; however, a strong Th17 signature was notable at the transcript and protein levels in murine CLE skin. The role of IL-17 in human DLE pathogenesis is controversial. IL-17–producing T cells have been identified in kidney and skin lesions of patients with lupus (33–35), with 1 study of patients with DLE identifying IL-17A as highly expressed in both serum and damaged skin tissue (36). However, subsequent studies of lesional DLE skin, using microarray transcriptomic analyses or T cell crawl-out methods quantifying IL-17A production after T cell stimulation, did not identify a dominant Th17 profile (22, 23). Furthermore, Th17 cells were not identified in T cell subset analyses from single-cell RNA-Seq studies of DLE skin (24, 32). Similarly, our human DLE transcriptomic analyses did not identify a Th17 signature compared with healthy skin. In fact, T cell–rich areas of DLE skin biopsy specimens expressed somewhat lower Th17 marker gene expression than did healthy skin. The cause of this discrepancy between the murine model of cutaneous lupus and human skin disease is not clear but could involve differences in skin commensals, which may alter T cell IL-17 production (37, 38). Nonetheless, if present, it is possible that even low amounts of IL-17 may contribute to skin tissue damage in DLE. If so, this may be driven in part by HIF-1 because it favors pathogenic Th17 differentiation by targeting the proteosomal degradation of Foxp3 to control the balance between Th17 and Treg cells (11). Whether this pathway plays a role in human skin disease remains to be determined.

The factors driving HIF-1 upregulation in DLE skin have not been fully elucidated. Unlike what we observed in murine lupus nephritis (4), its upregulation in skin-infiltrating T cells was not related to detectable local tissue hypoxia as measured by pimonidazole staining, which can detect moderate to severely hypoxic conditions with oxygen tensions below 10 mmHg (18). However, pimonidazole does not identify modest to moderate hypoxic conditions, with oxygen tensions between 10 and 19 mmHg (14), which may promote HIF-1 expression. Such conditions are likely present in lesional CLE skin due to tissue damage and increased metabolic demands of the inflammatory infiltrate, though more studies will be required to characterize the oxygen tension in inflamed skin. In addition, hypoxia-independent mechanisms may increase HIF-1 expression in CLE skin–infiltrating T cells. TLR and T cell receptor signaling stabilize HIF-1 under normoxic conditions (7, 8), and T cell activation further enhances its stabilization in T cells cultured under hypoxic conditions (8), suggesting the potential for synergy between hypoxia-dependent and independent mechanisms. HIF-1 also accumulates in skin keratinocytes in response to UV light, which is mediated by mROS (16). The metabolic stress signal mROS is induced upon T cell activation (8) and upregulates HIF-1 in lymphocytes (39). We thus hypothesize that HIF-1 upregulation is multifactorial and warrants additional investigation to more precisely identify the factors resulting in its upregulation in CLE skin–infiltrating T cells.

Taken together, our results indicate that increased HIF-1 expression in CLE skin–infiltrating T cells promotes skin tissue damage, and its inhibition abrogates development of disease. We hypothesize that, as in lupus nephritis, skin-infiltrating T cells upregulate HIF-1 after infiltrating the skin, resulting in reprogramming of the T cells to subtypes with heightened effector functions, ultimately causing tissue damage. The identification of HIF-1 as a novel therapeutic target for DLE warrants further investigation, particularly because selective inhibitors of HIF-1 are available and have been well tolerated in phase II clinical trials for cancers (40).

## Methods

### Mice.

All mice were housed in the pathogen-free facility in the Yale Animal Resources Center (Yale University). MRL/MpJ-Faslpr/J (MRL/lpr) mice were purchased from The Jackson Laboratory.

### Isolation of murine skin-infiltrating T cells.

Fresh murine skin samples of diseased skin were collected from the interscapular region of 20- to 22-week-old MRL/lpr mice immediately after sacrifice. Skin was scraped with a razor blade on ice to remove subcutaneous fat and minced as previously described (41). Minced skin was digested in RPMI (Corning) containing 500 μg/mL Liberase TL (Millipore Sigma, catalog 5401020001) and 10^4^ U/mL DNase I (MP Biomedicals) at 37°C on an orbital incubator for 70 minutes. Digested skin was mashed through a 70 μm filter (Falcon), washed with RPMI, filtered again through a 70 μm filter, and either stained immediately for flow cytometry and cell sorting or plated for in vitro stimulation experiments, followed by staining.

### In vivo pimonidazole labeling.

To detect regions of hypoxia in vivo, mice were injected with 80 mg/kg pimonidazole (Hypoxyprobe, HP-200 mg) 1.5 hours prior to sacrifice, as previously described (42). Mice designated for flow cytometric analysis were injected with anti–CD45.1-PE 5 minutes prior to sacrifice to label circulating cells. Fresh dorsal skin and spleen samples were collected for T cell isolation, flow cytometry, IF, and RNA analysis. Lesional and nonlesional interscapular skin was harvested to generate samples of approximately 1–2 cm^2^ and were processed for flow cytometry as above or immediately fixed with periodate-lysine-paraformaldehyde and subsequently embedded in OCT for IF studies or immediately fixed with 10% normal buffered formalin with subsequent embedding into paraffin for RNAScope experiments.

### Pharmacologic HIF-1 blockade.

Mice with no or early skin lesions were paired and treated with PX-478 (MedChemExpress, HY-10231) by oral gavage at the dosage of 5 mg/kg every 2 days for 4 weeks to inhibit HIF-1 in vivo, and control mice received an equivalent amount of PBS (4 mL/kg). Treatment was started at 16 weeks of age and ended at age 20 weeks. For single-cell RNA-Seq experiments, 20-week-old MRL/lpr mice were orally gavaged at the dose of 30 mg/kg daily for 5 days to block HIF-1 in vivo; control mice were gavaged with the same volume of PBS.

### Mouse skin assessment and histopathologic scoring.

Skin was assessed when mice were 20 weeks old (end of treatment). Photographs were taken and were stripped of identifiers such that gross skin lesion score could be assessed in a blinded manner. Gross skin lesions were then graded by 2 scorers on a scale of 0 to 3, with 0 indicating none, 1 indicating mild (snout and ears), 2 indicating moderate (snout, ears, and interscapular; <1 cm per lesion), and 3 indicating severe (snout, ears, and interscapular; >1 cm per lesion or severe clinical assessment) (43). The majority of mice received identical scores from both masked scorers, and all scores were within 1 point; the average dermatitis score is reported for any mouse that received nonidentical scores.

At 20 weeks of age (end of treatment), mice were sacrificed, and shaved interscapular skin samples were immediately collected, fixed in 10% buffered formalin, and paraffin-embedded. H&E-stained sections were scored in a blinded manner by 1 observer according to a semiquantitative scale of 0–2 for acanthosis, hyperkeratosis, interface (liquefaction), and inflammation (44), with total histopathologic disease score per mouse reported as the sum of these subscores.

### Flow cytometry and cell sorting and in vitro stimulation.

Skin tissue was processed as above. Spleen tissues were homogenized by crushing with the head of a 1 mL syringe in a Petri dish followed by straining through a 40 μm nylon filter (Falcon). Ammonium-chloride-potassium buffer was used for red blood cell lysis, and remaining cells were counted. Abs used for flow cytometry staining included anti–mouse CD3 (clone 17A2; BioLegend, catalog 100228) anti–mouse TCR-β (clone H57-597; BD, catalog 553170), anti–mouse CD4 (clone RM4-5; BioLegend, catalog 100548), anti–mouse CD8a (clone 53-6.7; BioLegend, catalog 100759), anti–human/mouse CD44 (clone IM7; eBioscience, catalog 47-0441-82), anti–mouse CD45 (clone 30-F11; BD, catalog 552848), anti–mouse CD45.1 (clone A20; Thermo Fisher Scientific, catalog 12-0453-82), anti–mouse CD45R (clone RA3-6B2; BD, catalog 562290), anti–mouse CCR6 (clone 140706; BD Horizon, catalog 564736), anti-pimonidazole FITC-Mab (clone 4.3.11.3; Hypoxyprobe, catalog HP6-200), LIVE/DEAD Fixable Aqua Dead Cell Stain Kit (Invitrogen, catalog L34966), anti–HIF-1α (clone H1alpha67; Novus Biologicals, catalog NB-100-105AF647), anti-RORγt (clone B2D; eBioscience, catalog 12-6981-82), anti–T-bet (clone eBio4B10; eBioscience, catalog 12-5825-82), anti–granzyme B (clone GB11; Invitrogen, catalog GRB05), anti–IFN-γ (clone XMG1.2; eBioscience, catalog 50-7311-82), and anti-IL17a (clone TC11-18H10.1; BioLegend, catalog 506910). All stained samples were analyzed using an LSRII Multilaser Cytometer at the Flow Cytometry Facility, Yale University, and were analyzed by FlowJo 10.7.1. Cytokine staining was performed after in vitro stimulation, during which freshly isolated skin and spleen cells were plated at a density of 1 × 10^6^ live cells/mL and stimulated with RPMI complete medium containing 50 ng/mL phorbol 12-myristate 13-acetate and 1 μg/mL ionomycin with Brefeldin A for 4 hours at 37°C. After surface marker staining, intracellular cytokine staining was performed with BD Cytofix/Cytoperm and perm/wash buffer.

### RNA-Seq.

Skin-infiltrating and splenic CD4^+^ and CD8^+^ T cells were isolated and sorted from lesional skin and spleens of 20-week-old MRL/lpr mice, as described above, and mRNA-isolated (RNeasy Plus Micro Kit; QIAGEN, catalog 74034). SMART-Seq v4 Ultra Low Input RNA Kit for Sequencing (TaKaRa, catalog 634893) was used to construct the sequencing library. Samples were sequenced on an Illumina HiSeq 2000 with 100 bp paired ends (Yale Center for Genome Analysis Core facility). Sequence alignment to mouse genome MM9 was done by using STAR 2.5.3 and TopHat 2.1.0 on the Partek Flow platform. The differentially regulated genes were analyzed by DESeq2 (45).

For single-cell RNA-Seq with simultaneous surface protein detection of CD4, CD8a, and CD45RB, lesional skin and spleens of 20-week-old MRL/lpr mice were harvested 1 day after treatment with either PBS or PX-478 at the dose of 30 mg/kg daily for 5 days. After preparation and staining of single-cell suspensions from skin, as described above, live CD45^+^ and CD45^–^ single cells were sorted and admixed at a 50:50 ratio. These cells and whole splenocytes, prepared as described above, were then stained for surface protein (Feature Barcoding Technology) per BioLegend protocol with 0.5 μg of each oligonucleotide-tagged TotalSeq-C Ab: anti–mouse CD4 (BioLegend, catalog 100571), anti–mouse CD8a (BioLegend, catalog 100785), and anti–mouse CD45RB (BioLegend, catalog 103321). Cells were then submitted immediately for 10× single-cell RNA-Seq at the Yale Center for Genome Analysis. The libraries were prepared by the Yale Center for Genome Analysis and sequenced by NovaSeq 6000. The sequenced files were aligned using standard 10x Genomics Pipeline, Cell Ranger, version 5.0.1. High-quality cells, defined as having at least 200 detected genes, with percentage of mitochondrial genes per cell lower than 10%, were used for downstream analysis, and cell clustering was done with Seurat, version 4.1.1. Cluster identities were determined using the cluster identity predictor (CIPR) package in *R* (46) and verified by manual review of cell type–specific transcripts by cluster. Single-cell GSEA was done by escape, version 1.6.0 (47), referencing the following gene sets: Th17 cell differentiation from the Kyoto Encyclopedia of Genes and Genomes 2021 (48), T Cell–Mediated Cytotoxicity from the Jax Mouse Genome Informatics Gene Ontology Project (GO:0001913) (49), and HIF1α-regulated genes derived from GSE35111 (20).

### GSEA.

GSEAs were performed using GSEA software (version 3.0) maintained by The Broad Institute (50).

### IF microscopy.

For murine skin IF samples, immediately after sacrifice, mouse skin tissues were fixed with periodate-lysine-paraformaldehyde fixative overnight. Samples were then dehydrated with 30% sucrose, embedded in OCT compound (Tissue-Tek), and stored at −80°C. Sections were cut at 7 μm and stained with the following Abs: anti–mouse CD4 (clone RM4-5; BioLegend, catalog 100547; 1:100), anti–mouse CD8a (clone 53-6.7; BioLegend, catalog 100747; 1:100), anti-pimonidazole mouse FITC- or DyLight 549-Mab (clone 4.3.11.3; Hypoxyprobe, catalog HP6-200 or HP7-200; 1:100), rabbit polyclonal anti–HIF-1α (GeneTex, catalog GTX127309; 5 μg/mL) or rabbit polyclonal isotype control (GeneTex, catalog GTX35035; 5 μg/mL), and Alexa Fluor 647–conjugated goat anti-rabbit secondary Ab (Thermo Fisher Scientific, catalog A-21244; 1:1,000). Confocal microscopy was conducted with a Leica SP8 (Figure 1 and Supplemental Figure 2) or SP5 (Supplemental Figure 3) laser-scanning confocal microscope at the Cell Imaging Core, Yale Stem Cell Center.

### RNAScope ISH.

The RNAScope 2.5HD RED assay kit (Advanced Cell Diagnostics) was used to perform ISH for RNA detection in human DLE and healthy skin samples as well as MRL/lpr skin samples collected from PX-478 treatment cohorts. Slides were prepared and hybridized with RNA probes according to the manufacturer’s instructions for FFPE samples. RNA probes included human and mouse *GZMB* (catalog 445971 and 49019) and *IL17A* (catalog 310931 and 319571), positive control peptidyl-prolyl isomerase B (*PPIB*) housekeeping gene (catalog 313901 and 313911), and negative control *DapB* soil bacteria enzyme (catalog 310043). Amplification and signal detection steps were performed according to kit instructions. Slides were then counterstained using hematoxylin and bluing reagent (Scigen catalog 23-730-614) and mounted with coverslips using Permount (Fisher Scientific catalog SP15-100).

IHC staining was performed on human DLE samples after RNA ISH using the RNAScope 2.5HD RED assay kit. A second round of antigen retrieval was performed in citrate buffer (Thermo Fisher Scientific, catalog 00500) for 30 minutes, and a peroxidase block was applied using 3% hydrogen peroxide (JT Baker, catalog JT-2186-01) for 30 minutes. Subsequent blocking and staining steps were executed according to the ImmPRESS HRP Reagent kit (catalog MP-7401) using normal horse serum. Slides were stained with primary anti–human CD3e (Cell Signaling Technology, catalog 85061; 1:150) at 4°C overnight and HRP horse anti-rabbit IgG secondary (ImmPRESS, catalog MP-7401) for 1 hour at room temperature. Signal was developed with DAB substrate (Vector Laboratories, catalog SK-4100) prior to counterstaining and being mounted with coverslips, as described above.

FISH-IF in human DLE samples was performed according to the integrated codetection protocol using the RNAScope Multiplex Fluorescent v2 Assay kit (Advanced Cell Diagnostics). Slides were prepared according to manufacturer’s instructions for FFPE samples using the RNA-Protein Co-detection Ancillary Kit (Advanced Cell Diagnostics, catalog 323180). Slides were incubated overnight with anti-CD3e primary Ab (clone SP7, Thermo Fisher Scientific, catalog MA5-14524; 1:50) and processed according to manufacturer’s instructions for integrated protein-RNA codetection. RNA probes included human *GZMB* (catalog 445971), positive control *PPIB* (catalog 320861), and *DapB* soil bacteria (catalog 320871). Amplification and signal detection steps were performed according to kit instructions for channel 1 and stained using Opal 570 (Akoya, catalog FP1488001KT; 1:1500). Slides were stained with Alexa Fluor 647–conjugated goat anti-rabbit secondary Ab (Thermo Fisher Scientific, catalog A-21244; 1:200), then counterstained using DAPI. Slides were mounted with coverslips with Prolong Gold Antifade (Thermo Fisher Scientific, catalog P36930) and imaged using the Leica SP8 Gated STED 3× super-resolution microscope at the Yale Center for Cellular and Molecular Imaging.

### RNA ISH quantification.

Slides stained by RNAScope 2.5HD RED assay kit were imaged using SPOT image software on a Zeiss Axioskop 40 light microscope under ×400 magnification. RNA staining was quantified using Qupath Bioimage analysis software, version 0.2.3, with the ImageJ Fiji version 2.10 extension. Quantification was performed using the bright-field image nucleus detection program StarDist (51) with the he_heavy_augment model (52). The threshold for positive staining was set at 1 dot per cell and each sample was manually checked for accurate classification of positive staining in a blinded fashion. The number of positive-staining cells was expressed as a percentage of the total cells in each high-powered field.

Slides stained by RNAScope Multiplex Fluorescence v2 Assay kit were quantified using HALO image analysis software (HALO 3.5.3577.214 and HALO AI 3.5.3577). Quantification was performed using the HALO FISH-IF algorithm (version 2.1.5) with the HALO Spatial Analysis module (version 3.5). T cells were identified by surface staining with CD3, and granzyme B–positive cells were identified with a threshold for positive staining set at 4 dots per cell. Samples were manually checked for accurate classification of positive staining. Spatial distribution of granzyme B–positive T cells was calculated by defining the DEJ or border of the hair follicle and analyzing the dermal infiltrate for granzyme B–positive CD3^+^ cells as a function of distance from the defined border, in 50 micron “bins,” from 0 to 300 or 350 microns away from the DEJ or hair follicle, respectively (Supplemental Figure 5, D and E). The number of granzyme B–positive staining CD3^+^ T cells was expressed as a percentage of the total CD3^+^ T cells in each of the 50 μm bins and was plotted as percentage of granzyme B–positive T cells as a function of distance (Supplemental Figure 5, B and C).

### NanoString GeoMx DSP.

Samples of DLE and control skin from cyst excisions from archived FFPE tissue were obtained from the Yale Dermatopathology biorepository. Sections of 5 μm were cut and placed on glass slide, with each glass slide including 3 samples from the same disease or control state, prior to shipping slides to NanoString for GeoMx DSP. The specific regions of interest (ROIs) for molecular profiling were then selected based on location of CD3^+^ staining.

ROIs were profiled using the GeoMx Digital Spatial Profiler (NanoString) (53). Slides were incubated with a multiplexed cocktail of RNA oligonucleotide probes with UV-photocleavable indexing oligonucleotides (Cancer Transcriptome Atlas) and 4 fluorescent markers (Syto83 at 500 μM for nuclei visualization; CD3-AF594 [Novus, catalog C3e/1308; 1:100]; CD8-647 [Novus, catalog SPM548; 1:200]; and PanCK-AF488 [Novus, catalog AE1/AE3; 1:500]). For RNA analysis, each ROI was a geometric shape approximately 300 μm in diameter with 3 CD3-rich ROIs selected per sample. ROIs were then exposed to UV illumination to cleave DNA oligonucleotides from the tissue. Cleaved oligonucleotides were collected through microcapillary aspiration and placed in a microwell plate. Oligonucleotides from each ROI were contained in separate wells. For Cancer Transcriptome Atlas, collected oligonucleotides were amplified using a forward primer and a reverse primer that serve as Illumina i5/i7, unique, dual-indexing sequences to index ROI identity. After purifying the PCR products with AMPure XP beads (Beckman Coulter), they were sequenced. Library purity and concentration were measured with a DNA Bioanalyzer chip (Agilent). Reads after sequencing were trimmed, merged, and aligned to retrieve the identity of probes. PCR duplicates and duplicate reads were removed, and the reads were converted to digital counts. The RNA-Seq saturation was sufficient and greater than 50%. After removing the outlier probes, the mean of the individual probe counts is considered the reported count value. Using GeoMX software (NanoString), 75% upper quartile (Q3) of the counts per ROI were selected after removing genes with 0 counts. The Q3 normalized counts were compared across the ROI and disease subtypes, using several approaches.

### Statistics.

Data were analyzed using the appropriate indicated statistical test (e.g., Student’s *t* test, Mann-Whitney test, Spearman correlation, or Kruskal-Wallis test) with Prism 8 (GraphPad Software). *P* < 0.05 was considered statistically significant.

### Study approval.

All murine experimental protocols were approved by the Yale IACUC (approval 2022-07801). Samples of human DLE and control healthy skin from excisions from archived FFPE tissue were obtained from the Yale Dermatopathology biorepository. Use of archived human FFPE tissue was approved by the Yale University Human Investigative Committee (approval 15010105235).

### Data availability.

The MRL/lpr bulk and single-cell RNA-Seq data are available in the National Center for Biotechnology Information Gene Expression Omnibus database under accession number GSE229407. The human DLE NanoString GeoMx Digital Spatial Profiling transcriptomics data are available on Mendeley Data (https://data.mendeley.com/datasets/ck9f9rkdvw/1). Values for all data points found in graphs are in the Supporting Data Values file.

## Author contributions

AJL, PMC, and JC conceptualized the study and wrote the manuscript; the methodology was devised by AJL, PMC, MDV, and RNK; AJL, PMC, MDV, RNK, JMM, JF, JG, and FIM conducted the investigations; AJL and MDV performed masked clinical scoring of murine skin lesions; JMM performed masked histopathologic scoring of murine skin lesions; JC reviewed and edited the manuscript and supervised the study; JC, AJL, and MV were responsible for funding acquisition; and JC and JMM provided resources.

## Supplementary Material

Supplemental data

Supporting data values

## Version 1 08/01/2023

In-Press Preview

## Version 2 08/22/2023

Electronic publication
