# Supplementary material for: HIF-1 regulates pathogenic cytotoxic T cells in lupus skin disease
Source: JCI Insight. 2023 Aug 22;8(16):e166076. doi: 10.1172/jci.insight.166076 (PMC10543720; doi:10.1172/jci.insight.166076)
Supplement: Supplemental data [file jciinsight-8-166076-s021.pdf]

Supplementary Materials for

HIF-1 regulates pathogenic cytotoxic T cells in lupus skin disease

Alicia J. Little, Ping-Min Chen, Matthew D. Vesely, Rahanna N. Khan, Jacob Fiedler, James Garritano, Fahrisa M. Islam, Jennifer M. McNiff, and Joe Craft

Correspondence: Joseph E. Craft; Yale University School of Medicine; 300 Cedar St, PO Box 20803; New Haven, CT 06520-8031; joseph.craft@yale.edu; and Alicia J. Little; Yale University School of Medicine; 300 Cedar St, PO Box 208059; New Haven, CT 06520-8059; alicia.little@yale.edu.

This PDF file includes:

Figure S1. Gating strategy for skin-infiltrating CD4$^+$ and CD8$^+$ T cells.
Figures S2. Skin-infiltrating T cells demonstrate HIF-1 expression by immunofluorescence.
Figure S3. Diseased MRL/lpr skin demonstrates pimonidazole-positive hypoxic regions in the dermis, but T cell infiltrates are not limited to areas of identified hypoxia.
Figures S4. 10X single-cell RNA sequencing identifies CD4$^+$ and CD8$^+$ T cells in MRL/lpr skin and spleen by transcript and surface protein expression.
Figure S5. Human DLE demonstrates expression of cytotoxic molecules including granzyme B in the DLE lymphocytic infiltrate in proximity to the dermoepidermal junction and hair follicle.

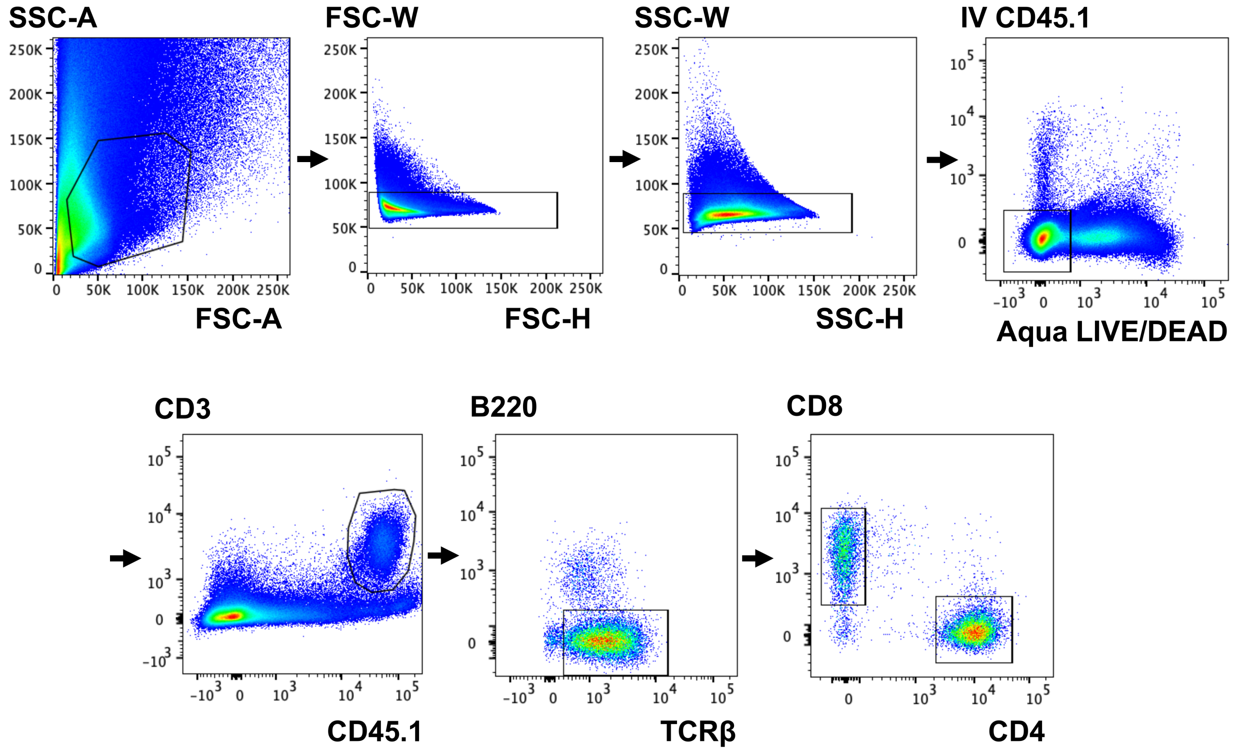

Figure S1. Gating strategy for skin-infiltrating CD4⁺ and CD8⁺ T cells. After defining single cell lymphocyte gates by forward and side scatter, T cell populations were determined using gates for live hematopoietic (CD45.1⁺) CD3⁺ TCRβ⁺ B220⁻ cells. Intravascularly (IV) injected CD45.1-PE was used to separate tissue-infiltrating from circulating CD4⁺ and CD8⁺ T cells.

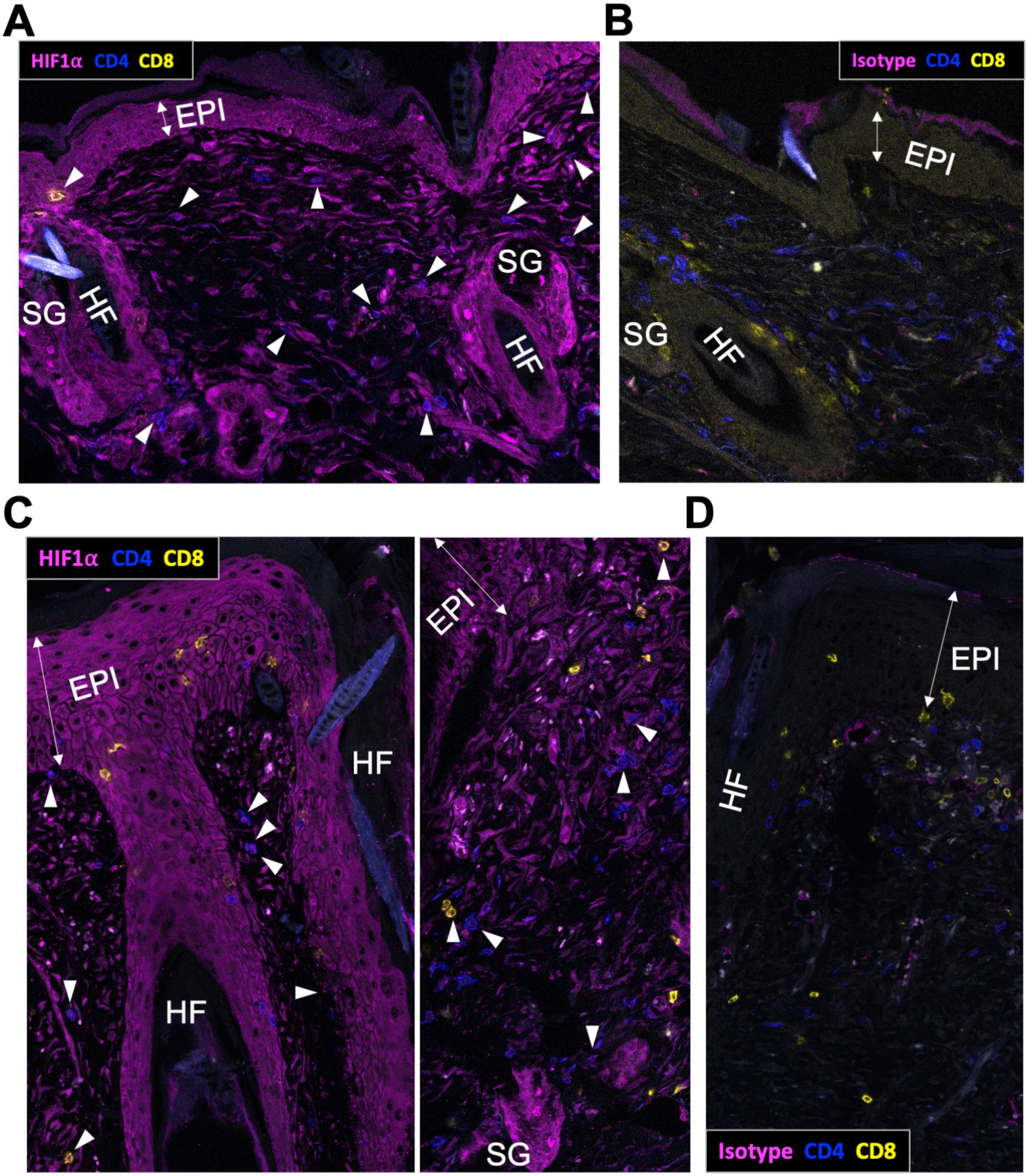

Figure S2. Skin-infiltrating T cells demonstrate HIF-1 expression by immunofluorescence. A, B. Immunofluorescence images of MRL/lpr diseased skin stained for HIF1a (A, C) or isotype control (B, D) (magenta), CD4 (blue), and CD8 (yellow). White arrow heads indicate HIF-1 expressing CD4+ or CD8+ T cells. Representative of 7 total sections from 2 mice (A, B and C, D are separate mice). EPI, epidermis. SG, sebaceous gland. HF, hair follicle.

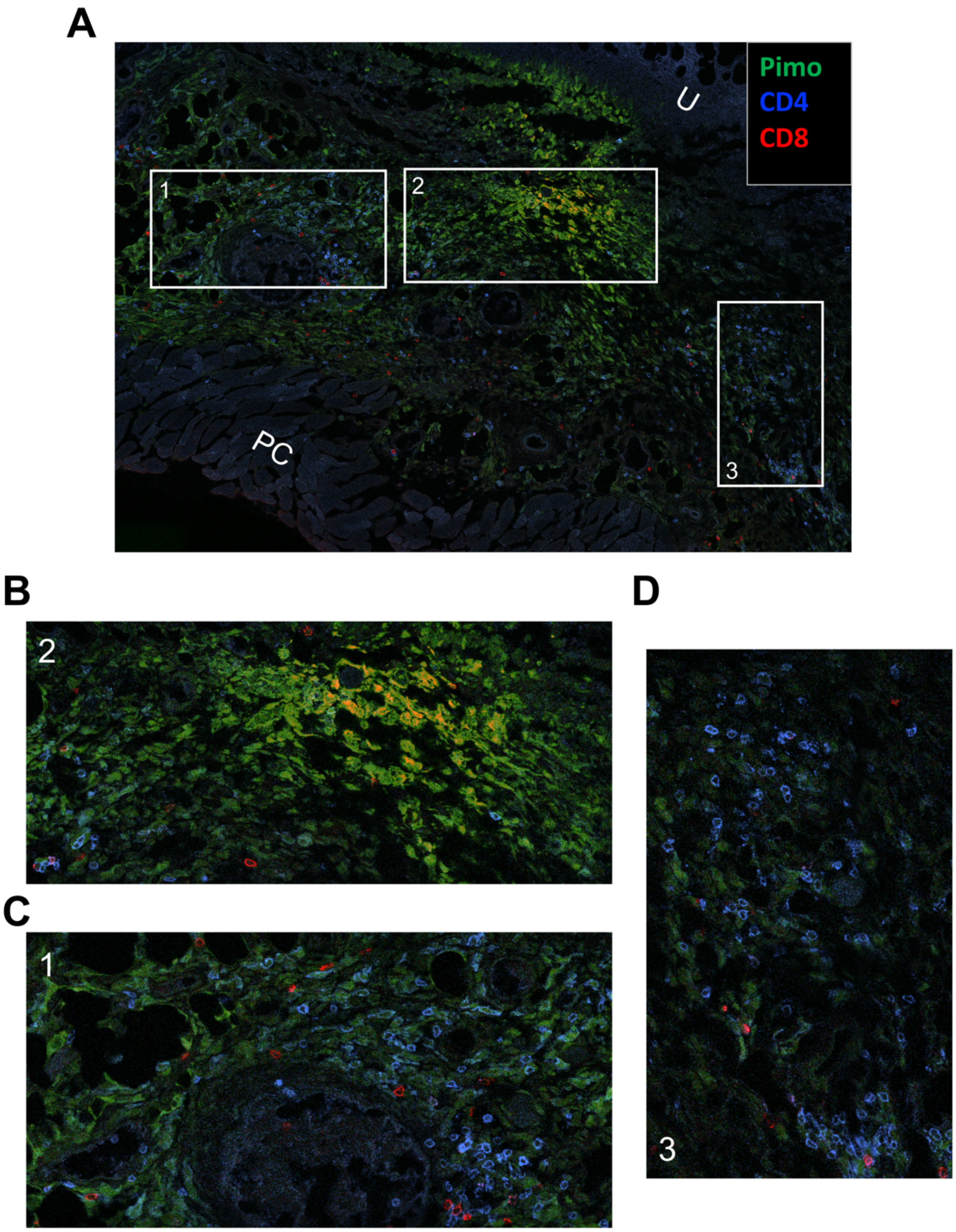

Figure S3. Diseased MRL/lpr skin demonstrates pimonidazole-positive hypoxic regions in the dermis, but T cell infiltrates are not limited to areas of identified hypoxia. A. Immunofluorescence images of MRL/lpr diseased skin stained for pimonidazole (pimo, green), CD4 (blue), and CD8 (red). U, ulcerated epidermis. PC, panniculus carnosus muscle. Magnified insets are outlined and numbered. **B-D**. Magnified insets of high (B), medium (C), and low (D) pimonidazole-staining regions of the dermis, demonstrating $CD4^+$ and $CD8^+$ T cell infiltrates. Representative of n = 3 mice.

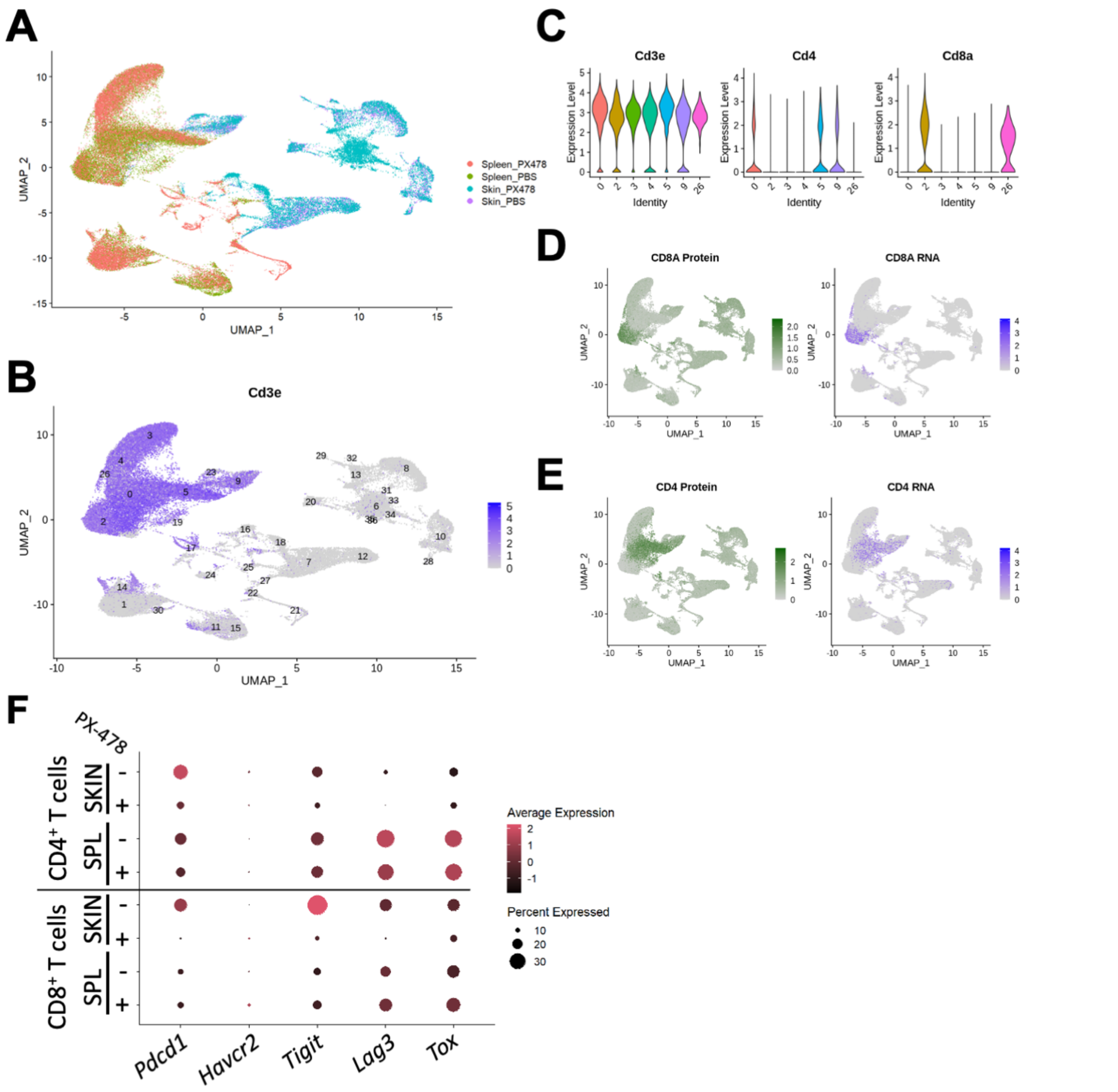

Figure S4. 10X single-cell RNA sequencing identifies $CD4^+$ and $CD8^+$ T cells in MRL/lpr skin and spleen by transcript and surface protein expression. A. Combined UMAP plot of single-cell RNA sequencing data derived from cells isolated from the skin or spleen of 20-week-old MRL/*lpr* mice after 5 days of treatment with either PBS (vehicle) or selective HIF1α inhibitor PX-478 (treated), colored by organ of origin (skin vs spleen) and treatment group (PBS vs PX-478). **B**. Combined UMAP plot shaded to demonstrate normalized expression level of Cd3e, which highlights clusters identified as T cells (0, 2, 3, 4, 5, 9, 26) for further analysis. Cluster identities were determined using the CIPR (cluster identity predictor) package in *R* and confirmed by manual review of cell-type specific transcripts by cluster (see *Methods*). **C.** Violin plot demonstrating *Cd3e*, *Cd4*, and *Cd8a* transcript expression by cluster, highlighting clusters identified as $CD4^+$ T cells (0, 5, 9), $CD8^+$ T cells (2, 26) and double-negative T cells (3, 4). **D.** Combined UMAP plots shaded to demonstrate normalized expression level of CD8A protein (left) or transcript (right). **E.** Combined UMAP plots shaded to demonstrate normalized expression level of CD4 protein (left) and transcript (right). **F.** Dot plot demonstrating normalized expression level (average expression) and percent of cells expressing selected exhaustion-associated genes in $CD4^+$ (top) or $CD8^+$ (bottom) T cell clusters, separated by organ of origin (skin vs spleen) and treatment group (PBS vs PX-478). SPL, spleen.

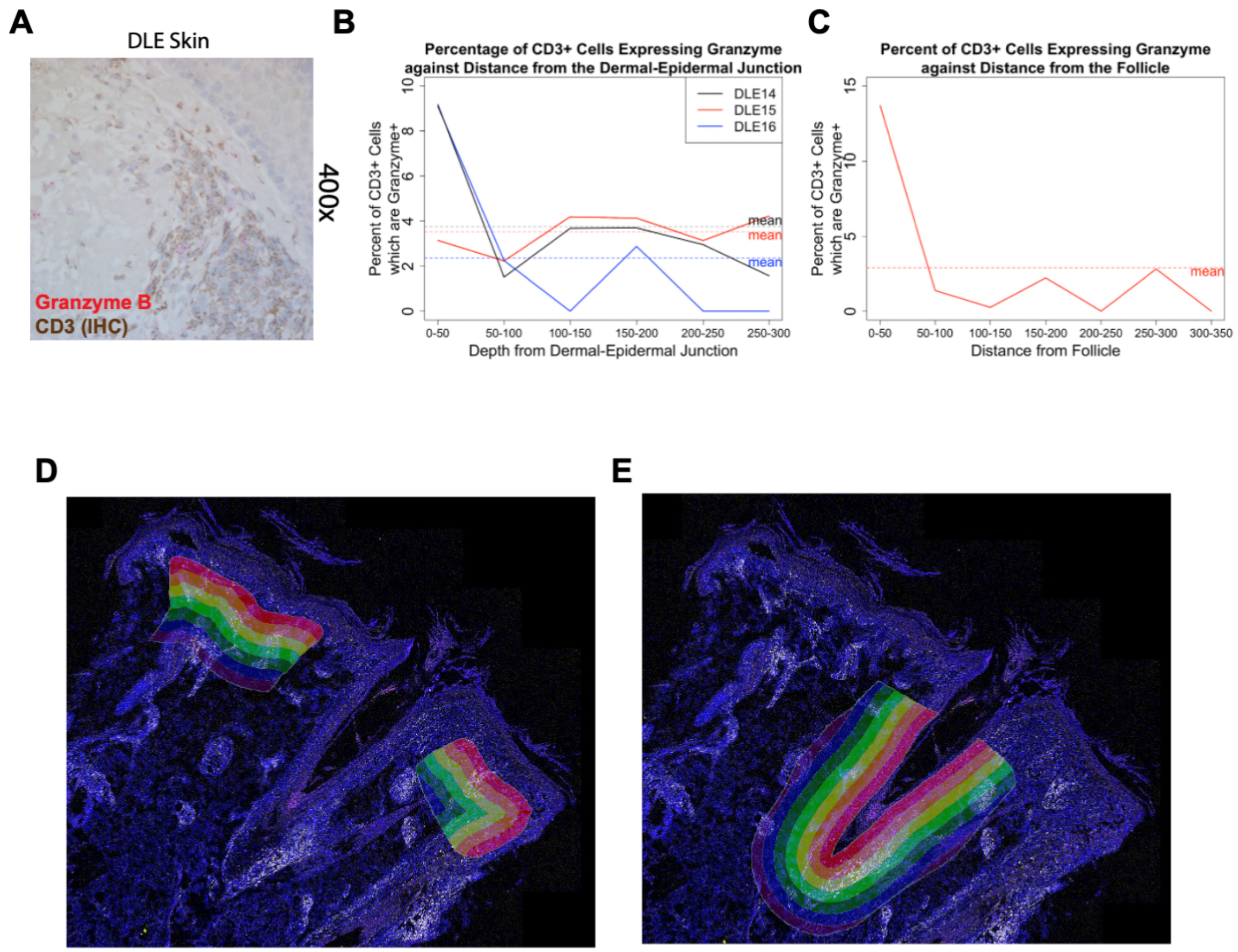

Figure S5. Human DLE demonstrates expression of cytotoxic molecules including granzyme B in the DLE lymphocytic infiltrate in proximity to the dermoepidermal junction and hair follicle. A. Representative image of DLE skin demonstrating *GZMB* RNA expression (red) by RNA ISH and CD3 protein expression by IHC (brown) in FFPE DLE skin detected by RNAscope-RED Assay^{TM} at 400x original magnification. **B, C.** Percentage of *GZMB*+ CD3+ T cells as a function of distance from the dermoepidermal junction (B) or hair follicle (C) as detected in FFPE DLE skin by RNAscope Multiplex Fluorescent V2 Assay^{TM} at 400x original magnification. Quantified with HALO (see *Methods*). **D, E**. Representative images used for spatial analysis with colored overlay indicating the 50 um bands used for spatial analysis, ranging from 0-350 um from the dermoepidermal junction (D) or hair follicle (E). Representative of n = 3 cases of DLE for dermoepidermal junction (B) or 1 case for hair follicle (C) due to absence of hair follicles amenable to distance analysis in other DLE cases.
